# Systemic endotoxinemia as a probable factor in reducing the treatment effectiveness of endogenous psychosis

**DOI:** 10.1192/j.eurpsy.2021.1260

**Published:** 2021-08-13

**Authors:** S. Zozulya, I. Otman, I. Oleichik, I. Anikhovskaya, M. Yakovlev, T. Klyushnik

**Affiliations:** 1 Laboratory Of Neuroimmunology, Mental Health Research Centre, Moscow, Russian Federation; 2 Clinical Department Of Endogenous Mental Disorders And Affective States, Mental Health Research Centre, Moscow, Russian Federation; 3 Laboratory Of Systemic Endotoxinemia And Shock, Institute of General Pathology and Pathophysiology, Moscow, Russian Federation

**Keywords:** endogenous psychosis, inflammatory markers, treatment effectiveness, systemic endotoxemia

## Abstract

**Introduction:**

Inflammation is an important factor in the pathogenesis of endogenous psychosis. An inducer of inflammatory reactions can be endotoxin aggression of intestinal origin.

**Objectives:**

To determine the level of inflammation markers and indicators of systemic endotoxinemia in blood of patients with endogenous psychosis in relation to assessment of the treatment effectiveness.

**Methods:**

25 patients with endogenous psychosis (F20, F25) were examined before and after treatment. The control group consisted of 25 healthy people. The activity of inflammatory markers - leukocyte elastase, α1-antitrypsin, antibodies to S-100B, and indicators of systemic endotoxinemia – endotoxin concentration and antiendotoxin immunity activity were measured in blood serum. The treatment effectiveness was assessed by the dynamics of inflammatory markers.

**Results:**

Based on the results of determining the studied parameters before treatment, all patients were divided into two groups. In the 1st group (6 patients, 24%), an increase of inflammatory markers activity and high concentration of endotoxin in the blood serum were revealed (p<0,001, p<0,05, respectively). In the 2nd group (19 patients, 76%), only activation of inflammatory reactions (p<0,001) was detected. After therapy in the 1st group of patients, there was no positive dynamics of all studied markers, which indicated an active course of the pathological process. In the 2nd group, the normalization of inflammatory markers was shown (p<0,05), which corresponded to the formation of remission.

**Conclusions:**

The results indicate that endotoxic aggression contributes to reduction of the effectiveness of endogenous psychosis therapy and can be considered as an additional therapeutic target.

